# Suboptimal Follow-Up on HIV Test Results among Young Men Who Have Sex with Men: A Community-Based Study in Two U.S. Cities

**DOI:** 10.3390/tropicalmed7070139

**Published:** 2022-07-19

**Authors:** Ying Wang, Jason Mitchell, Chen Zhang, Lauren Brown, Sarahmona Przybyla, Yu Liu

**Affiliations:** 1 Department of Public Health Sciences, School of Medicine and Dentistry, University of Rochester Medical Center, Rochester, NY 14642, USA; ying_wang@urmc.rochester.edu; 2 Department of Health Promotion and Disease Prevention, Stempel College of Public Health and Social Work, Florida International University, Miami, FL 33199, USA; jamitche@fiu.edu; 3 School of Nursing, University of Rochester Medical Center, Rochester, NY 14642, USA; chen_zhang@urmc.rochester.edu; 4 Department of Psychiatry and Behavioral Sciences, Meharry Medical College, Nashville, TN 37208, USA; llbrown@mmc.edu; 5 Department of Medicine, Vanderbilt University Medical Center, Nashville, TN 37232, USA; 6 Department of Community Health and Health Behavior, School of Public Health and Health Professions, University at Buffalo, Buffalo, NY 14214, USA; mona@buffalo.edu

**Keywords:** young men who have sex with men, HIV prevention, HIV testing, follow-up on HIV test results, behavioral determinants, psychosocial determinants, United States

## Abstract

Frequent HIV testing and knowledge of HIV serostatus is the premise before timely access to HIV prevention and treatment services, but a portion of young men who have sex with men (YMSM) do not always follow up on their HIV test results after HIV testing, which is detrimental to the implementation of HIV prevention and care among this subgroup. The comprehensive evaluation of factors associated with inconsistent follow-up on HIV test results may inform relevant interventions to address this critical issue among YMSM. To this end, we conducted a cross-sectional study in Nashville, Tennessee and Buffalo, New York from May 2019 to May 2020 to assess demographic, behavioral, and psychosocial correlates of inconsistent follow-up on HIV test results among YMSM. Of the 347 participants, 27.1% (*n* = 94) reported inconsistent follow-up on their HIV test results. Multivariable logistic regression showed that inconsistent follow-up on HIV test results was positively associated with condomless receptive anal sex, group sex, recreational drug use before or during sex, internalized homophobia, and stress; while negatively associated with housing stability, social support, and general resilience. Future HIV prevention intervention efforts should target these modifiable determinants to enhance the follow-up on HIV test results among YMSM.

## 1. Introduction

Approximately 1.2 million people aged 13 years and older in the United States (U.S.) were estimated to be living with HIV, including 158,000 undiagnosed HIV infections. Over 60% of 36,801 new HIV diagnoses in 2019 were among men who have sex with men (MSM), of whom approximately two-thirds were between 13–34 years of age [1]. Prior literature has identified a plethora of behaviors that may predispose young men who have sex with men (YMSM) at higher risk of HIV acquisition, including condomless anal sex, substance use, and multiple sex partners [2,3,4]. Studies have reported YMSM may be more prone to experiencing higher levels of social stigma and mental health burdens, while also lacking the necessary experience in finding and accessing HIV prevention services (e.g., HIV testing, pre-exposure prophylaxis [PrEP]) [5,6,7,8]. These findings underscore that YMSM represent the sex and gender minority subgroup of the highest priority for targeted HIV prevention interventions in the U.S.

Accurate knowledge of one’s HIV serostatus through frequent HIV testing is important to help curtail the HIV epidemic among YMSM in the U.S. [9,10]. Knowledge of one’s HIV serostatus requires being tested and receiving the results as a precursor before any linkage to evidence-based HIV prevention-care services can occur, including antiretroviral therapy (for individuals with HIV positive diagnosis) and PrEP care (for high-risk individuals who are HIV negative) [11,12]. Furthermore, knowledge of one’s HIV serostatus as seropositive accompanies counseling and linkage to appropriate care services, which has been shown to increase awareness of and engagement in risk-reduction strategies, including treatment adherence—all important steps to prevent the onward transmission of HIV [11,13]. 

Given the importance of timely knowledge of one’s HIV serostatus, an increasing evidence base of HIV prevention interventions have been designed and implemented to improve regular HIV testing uptake among YMSM in the U.S. [14,15,16,17,18]. However, the uptake of HIV testing is not equivalent to the knowledge of HIV test results. Studies have shown that MSM may experience low rates of follow-up on their HIV test results [19,20]. For instance, data from a randomized controlled trial to improve HIV testing among MSM in the U.S. showed that only 32% of intervention participants who requested HIV testing kits followed up on their test results (vs. 0% in the control group) [21]. Studies among MSM in other countries found that 5–11% of MSM had failure-to-return for HIV test results [19,22]. Prior literature reveals significant correlates of failure-to-return for HIV test results, including perceived risk of HIV infection, inconsistent condom use, and certain demographic characteristics such as male gender, young age, unemployment, rural residence, and low educational attainment [19,23,24,25,26]. Although these studies provide salient insights into factors associated with failure-to-return for HIV test results, few studies assessed relevant psychosocial determinants and their effects on HIV prevention uptake. Furthermore, despite the increasing number of interventions to enhance HIV testing among YMSM in the U.S., few emphasized the failure-to-return challenge and methods for increasing follow-up on test results. Moreover, no study to date has investigated the prevalence and factors associated with failure-to-return for HIV test results among YMSM in the U.S.

Comprehensive evaluation of failure-to-return for HIV test results and associated factors may inform HIV prevention efforts to address this critical issue among YMSM. We conducted a community-based study with YMSM in two U.S. cities to assess: (1) the prevalence of inconsistent (i.e., not always) follow-up on HIV test results, (2) demographic, behavioral, and psychosocial correlates, and (3) evaluate the relationship between inconsistent follow-up on HIV test results and various HIV prevention measures, including HIV testing, PrEP uptake, and condom use self-efficacy.

## 2. Materials and Methods

### 2.1. Procedures

The present quantitative, cross-sectional analysis comes from an HIV prevention study that was conducted in Nashville, Tennessee and Buffalo, New York between May 2019 and May 2020. The HIV prevention study investigated potential HIV risk factors and prevention outcomes among YMSM living in these two cities; findings on these relevant topics have previously been reported [5,27]. All procedures and related activities for the HIV prevention study and present analysis were approved by the Institutional Review Boards at the University of Rochester and University at Buffalo, and all participants provided informed consent.

A combination of a convenience sampling strategy, along with online and offline approaches, were used to recruit potential participants. Offline recruitment included peer referrals, flier distribution, and field outreach (e.g., at local LGBT events, sexual health centers, or gay bars). Online recruitment involved advertisements on social media sites used by YMSM (e.g., Instagram and Facebook). 

### 2.2. Participant and Data Collection

Eligibility criteria for the HIV prevention study and present analysis required participants to: (1) be between the ages of 18 and 35 years old; (2) report living in Nashville, TN or Buffalo, NY; (3) understand and read English; (4) self-report having engaged in sexual behaviors (e.g., oral and/or anal sex) with another male in the prior 12 months, or self-identify as gay, bisexual, or other cis-gender men; and (5) self-report negative or unknown HIV serostatus at the time of participation. Individuals who did not meet all inclusion criteria were excluded from participating. 

Additional details about the study procedures have been reported elsewhere [27]. In short, data were collected via a 40-min, self-administered survey delivered through Research Electronic Data Capture (REDCap). Participants could either complete the survey on site (i.e., local sexual health centers) or request a survey link from study staff to complete it at their preferred time and location. A $35 gift card was provided to each participant who completed the survey to acknowledge his effort for participating in our study.

### 2.3. Measures

The dependent variable, follow-up on HIV test results, was assessed by the item, “How often have you made sure you know the test result after each test you took?” Response options (never, rarely, sometimes, often times, every time) were dichotomized into consistent (i.e., always—“every time”) follow-up vs. inconsistent (i.e., not always—all other responses) follow-up. 

We also collected data on four broad categories: (1) sociodemographic characteristics (i.e., age, race/ethnicity, education, employment, annual personal income, health insurance coverage, sexual orientation, HIV risk perception, disclosure to healthcare providers (HCPs), and primary venues for finding sex partners); (2) behavioral factors including (a) recent (past six months) sexual behaviors (condomless receptive and insertive anal sex, group sex, condomless sex with HIV-positive men, and alcohol and recreational drug use before or during sex) and (b) substance use behaviors (tobacco use, binge drinking, and recreational drug use in the past 12 months); (3) psychosocial factors (i.e., housing stability, food insecurity [28], social support [29], general resilience [30], internalized homophobia [31], suicidal thoughts/behaviors [32], HIV-related stigma [33], loneliness [34], anxiety [35], depression [36], and stress [37]); and (4) HIV prevention measures (PrEP awareness, ever PrEP use, HIV home testing in the past 3, 6, 12 months and ever, HIV testing confidence [38], and condom use self-efficacy [39]).

### 2.4. Statistical Analyses

Continuous variables were summarized as means (standard deviations) and categorical variables were summarized as frequencies (percentages). Differences between consistent and inconsistent follow-up on HIV test results were compared using t-tests for normally distributed continuous variables, Mann–Whitney *U* tests for non-normally distributed continuous variables, or chi-square tests for categorical variables. Separate logistic regression models were built to assess correlates of inconsistent follow-up on HIV test results with each aforementioned behavioral and psychosocial factor as the primary independent variable, respectively. Each model individually adjusted for age, education, annual personal income, and sexual orientation. These variables were selected because they were considered a priori to be important predictors of knowledge of test results [23,25,40] and presented a significant association with the outcome in bivariate analyses (i.e., *t*-tests, chi-square tests). In order to assess the impact of inconsistent follow-up on test results on uptake of HIV prevention measures, we additionally developed separate logistic regression models adjusting for the aforementioned confounders to estimate adjusted odds ratio (aOR) and 95% Confidence Interval (CI) for each HIV prevention measure.

## 3. Results

Of 415 YMSM enrolled in the study, 347 completed the questionnaire and were included for analyses. Table 1 presents participants’ sociodemographic characteristics by the status of HIV test result follow-up. Most participants were 25–35 years of age (57.6%), Black or African American (60.2%), employed (70.0%), insured (81.3%), had above high school education (79.6%), earned less than $40,000 annual income (79.5%), self-identified as gay (76.9%), perceived high risk of HIV infection (78.1%), disclosed sexual orientation or same sex behaviors to HCPs (76.4%) and reported social media apps as a primary venue for finding sex partners (66.6%). In the analytic sample, 27.1% (*n* = 94) did not always follow up on their HIV test results. Educational level, age, annual income, health insurance, sexual orientation, disclosure of sexual orientation to HCPs, and venues to find sexual partners were significantly (*p* < 0.05) associated with inconsistent follow-up on HIV test results.

Table 2 shows descriptive statistics of behavioral risk factors and multivariable analyses of associations between each factor and inconsistent follow-up on HIV test results. Compared to YMSM with consistent knowledge of test results, those who did not consistently follow up on test results showed an overall higher prevalence across all risk behaviors, including recent condomless receptive and insertive anal sex, group sex, condomless sex with HIV-positive men, recreational drug use before or during sex, and substance use (e.g., tobacco use and binge drinking). After adjustment for age, education, income and sexual orientation, inconsistent follow-up on HIV test results was significantly associated with condomless receptive anal sex (aOR = 2.05; 95% CI, 1.13–3.69), group sex (aOR = 2.03; 95% CI, 1.12–3.68), and recreational drug use before or during sex (aOR = 1.82; 95% CI, 1.05–3.14).

Associations of psychosocial determinants and follow-up on HIV test results are presented in Table 3. YMSM who consistently followed up on their test results reported higher levels of housing stability, social support and general resilience, and had lower prevalence of psychosocial issues, including internalized homophobia, HIV related stigma, loneliness, anxiety, depression, and stress. Multivariable logistic regression analyses showed that lack of consistency in follow-up on test results was negatively associated with housing stability (aOR = 0.87; 95% CI, 0.80–0.96), social support (aOR = 0.98; 95% CI, 0.97–0.99), and general resilience (aOR = 0.96; 95% CI, 0.94–0.99), and positively associated with internalized homophobia (aOR = 1.08; 95% CI, 1.02–1.15) and stress (aOR = 4.49; 95% CI, 1.82–11.12).

Table 4 shows results from logistic regression analyses for associations of follow-up on HIV test results and HIV prevention outcomes. YMSM with inconsistent follow-up on their HIV test results were associated with lower levels of engagement along the PrEP care continuum including PrEP awareness (aOR = 0.37; 95% CI, 0.17–0.79) and ever PrEP use (aOR = 0.82; 95% CI, 0.44–1.55). Lack of consistency in follow-up on test results was also negatively associated with HIV testing confidence, condom use efficacy, and HIV testing in the past 3, 6, and 12 months.

## 4. Discussion

The present analysis extends the literature about YMSM’s knowledge of HIV test results by estimating the prevalence of their inconsistent follow-up on HIV test results and assessing potential correlates from a convenience sample residing in two U.S. cities. Our study found that more than one-quarter of YMSM did not consistently follow up on their every HIV test result, a finding that is 15.6% higher than reported in other studies of MSM [22]. The difference may be explained by predominantly young participants in the present study, which was a risk predictor of unawareness of HIV test results [41,42,43]. Our study further identified the negative association of inconsistent follow-up on HIV test results with HIV prevention outcomes, including HIV testing frequency/confidence, PrEP awareness/uptake, and condom use self-efficacy. The positive impact of knowledge of HIV serostatus on initiation of HIV treatment and adoption of safer behaviors (e.g., condom use) has been well documented in the literature [44], indicating that knowledge of HIV test results may be an important facilitator of the uptake of HIV prevention measures as well as a modifiable target for future HIV prevention efforts. Despite an increasing body of interventions designed to improve HIV testing uptake and repeat testing in recent years, few of them were attentive to post-test follow-up [18,21,45]. Therefore, future HIV prevention efforts may be improved by incorporating education about the importance of follow-up on one’s HIV test results.

Our study found that YMSM who inconsistently followed up on their test results had increased odds of sexual behaviors that may increase the possibility of acquiring HIV, namely engaging in condomless receptive anal sex, group sex, and using recreational drugs before or during sex. The association between engaging in these behaviors and one’s knowledge of HIV test results remains inconsistent in the literature. Some studies show the involvement of risky sexual behaviors (e.g., multiple sexual partners and no condom use at last sex) was associated with higher odds of follow-up on HIV test results [19,22,41,46], whereas other studies found that those with inconsistent condom use were less likely to return for HIV test results compared to those with consistent condom use [22,41]. The significant association of risky sexual behaviors and inconsistent follow-up on test results observed in our study could be explained by self-perception of low risk for HIV infection, fear of positive results, and participant age (as younger than 35 years of age is a group least likely to follow up on test results) [19,23,41,42]. Further investigation into the predictive role of risk behaviors in awareness of HIV test results is warranted. Finally, given the mounting evidence of onward transmissions caused by risky sexual behaviors among YMSM [47,48], future intervention efforts should emphasize both conventional sexual health education and sexual health communication including HIV status disclosure to sexual partners. 

In terms of psychosocial correlates, YMSM with high levels of stress were 4.49 times more likely to have inconsistent follow-up on HIV test results. Both the Vulnerability–Stress–Adaptation Model and minority stress theory maintain that MSM experience disproportionate chronic stressors, including internalized homophobia, stigma, prejudice, and discrimination [49,50]. People who experience higher stress levels tend to devalue the long-term benefits of HIV prevention behaviors [14], which may explain the lower engagement in HIV testing as well as follow-up on test results. Fortunately, current HIV prevention programs have employed multiple strategies (e.g., facilitated discussion and provision of community support) to raise awareness of stress among MSM and teach positive coping strategies [15,49]. Our study also contributes to future intervention efforts by identifying an association between general resilience and consistent follow-up on HIV test results among YMSM. Resilience has been previously linked to greater engagement in HIV prevention behaviors, and a decreased risk of condomless sex and mental health burdens among MSM [4,27,51]. Resilience was also observed to mediate the association of stress with risky sexual behaviors [50]. These results, along with the present study, suggest that resilience may be an important modifiable psychological target in future HIV intervention programs.

When evaluating the impact of social determinants of health on knowing test results, our study identified that housing stability and social support were both positive predictors of consistent follow-up on test results among YMSM. Defined as “structural determinants and conditions in which people are born, grow, live, work, and age” [52], social determinants are thought to be the root cause of health disparities [53,54] and have been linked to lower engagement in HIV prevention behaviors, including HIV testing, follow-up on test results, and PrEP adoption [42,46,55,56,57]. Qualitative studies further revealed that suboptimal uptake of HIV prevention services among people with negative social determinants of health could be explained by lack of transportation, shortage of HIV care providers in the neighborhood, lack of comprehensive HIV education, and poor social support [58,59]. The positive association of lack of consistency in follow-up on HIV test results with psychosocial adversities observed in our study (e.g., internalized homophobia, unstable housing, and food insecurity) also provides insights into the potential effects of different testing modalities on knowledge of test results. First, HIV self-testing may have the potential to help reduce the stigma and discrimination associated with traditional venue-based testing settings (e.g., perception or actuality of HCPs’ stigmatizing attitudes/behaviors toward patients, lack of confidentiality, and sexual orientation disclosure) by providing the individual with a safe, private environment to conduct the test and interpret the result [18,60,61]. Second, HIV self-testing may also help minimize the aforementioned logistic barriers that may prevent some individuals from going to HIV testing sites, including returning to them for a test result. For traditional facility-based HIV testing, offering a variety of ways to provide the test results (e.g., telephone, text message, mobile app or email) may help lessen YMSM who either do not receive or inconsistently receive their results. 

To the best of our knowledge, this is one of the few studies to examine sociodemographic, behavioral and psychosocial correlates of YMSM who never-to-inconsistently follow up on their HIV test results in two U.S. cities. However, limitations to the present analysis exist. As a cross-sectional study, the temporal relationships between the knowledge of HIV test results and the assessed correlates cannot be established. The generalizability of the findings to other YMSM throughout the U.S. must be cautioned, as the data and findings come from a convenience sample based out of two U.S. cities. Despite the use of computer-assisted self-interview, data collected by a self-administered questionnaire may be subject to recall and social desirability bias. We operationalized the study outcome as a binary variable due to the sparse response rate in some categories (i.e., never, rarely, sometimes, often times). We acknowledge that participants who never followed up on test results may differ from those who often knew the test results in behavioral/psychosocial factors. Therefore, the assessed determinants should be interpreted with caution and warrant more nuanced studies in the future. Finally, follow-up on test results relative to testing modality (e.g., HIV self-testing vs. clinic-based testing vs. community-based testing) was not assessed in the study and presents an avenue to be explored in future research.

## 5. Conclusions

Our study shows that 27.1% of YMSM were not consistently aware of the result after each HIV test experience. We identified negative associations between inconsistent follow-up on test results and HIV prevention outcomes, indicating that knowledge of HIV test results may be an important facilitator of the uptake of HIV prevention measures. Our study also documents significant correlates of inconsistent follow-up on HIV test results, including risky sexual behaviors (condomless receptive anal sex, group sex, and recreational drug use before or during sex) and psychosocial factors (housing stability, social support, general resilience, internalized homophobia, and stress). Despite an increasing body of interventions to enhance the uptake of HIV testing in recent years, few of them were attentive to post-test follow-up, suggesting that future HIV prevention efforts may be improved by targeting these modifiable determinants to enhance follow-up on HIV test results among YMSM.

## Figures and Tables

**Table 1:** Sociodemographic characteristics by the status of HIV test result follow-up among young men who have sex with men in two U.S. cities (N = 347).

Characteristics	Total (N = 347) *n* (%)	Not Always (N = 94) *n* (%)	Always (N = 253) *n* (%)	*p* Value
Age				0.0005
<25	147 (42.4)	54 (57.4)	93 (36.8)	
≥25	200 (57.6)	40 (42.6)	160 (63.2)	
Race				0.1716
White	109 (31.4)	30 (31.9)	79 (31.2)	
Black or African American	209 (60.2)	52 (55.3)	157 (62.1)	
Other ^a^	29 (8.4)	12 (12.8)	17 (6.7)	
Education				<.0001
High school diploma or less	71 (20.5)	34 (36.2)	37 (14.6)	
Some college	138 (39.8)	38 (40.4)	100 (39.5)	
College degree or higher	138 (39.8)	22 (23.4)	116 (45.8)	
Employment				0.0809
Employed	243 (70.0)	61 (64.9)	182 (71.9)	
Unemployed	51 (14.7)	12 (12.8)	39 (15.4)	
Student	53 (15.3)	21 (22.3)	32 (12.6)	
Income				0.0109
Less than $20,000	144 (41.5)	49 (52.1)	95 (37.5)	
$20,000 to less than $40,000	132 (38.0)	24 (25.5)	108 (42.7)	
$40,000 or more	71 (20.5)	21 (22.3)	50 (19.8)	
Insurance				0.0094
No	65 (18.7)	26 (27.7)	39 (15.4)	
Yes	282 (81.3)	68 (72.3)	214 (84.6)	
Sexual orientation				0.0002
Gay/homosexual	267 (76.9)	60 (63.8)	207 (81.8)	
Heterosexual	32 (9.2)	18 (19.1)	14 (5.5)	
Bisexual	48 (13.8)	16 (17.0)	32 (12.6)	
HIV risk perception				0.0543
High/very high	271 (78.1)	80 (85.1)	191 (75.5)	
No/low	76 (21.9)	14 (14.9)	62 (24.5)	
Sexual orientation disclosure to healthcare professionals				<0.0001
No	82 (23.6)	37 (39.4)	45 (17.8)	
Yes	265 (76.4)	57 (60.6)	208 (82.2)	
Venues to find sex partners				<0.0001
Gay-frequented venues	61 (17.6)	9 (9.6)	52 (20.6)	
Internet/website	55 (15.9)	29 (30.9)	26 (10.3)	
Social media app	231 (66.6)	56 (59.6)	175 (69.2)	

[^a^] Including Hispanic/Latino, Asian, and not sure.

**Table 2:** Association of risk behaviors and inconsistent follow-up on HIV test results among young men who have sex with men in two U.S. cities (N = 347).

Characteristics	Total (N = 347) *n* (%)	Not Always (N = 94) *n* (%)	Always (N = 253) *n* (%)	aOR (95% CI) ^a^
Condomless receptive anal sex with a man in the past six months				
No	146 (42.9)	33 (36.7)	113 (45.2)	Reference
Yes	194 (57.1)	57 (63.3)	137 (54.8)	2.05 (1.13, 3.69)
Condomless insertive anal sex with a man in the past six months				
No	137 (40.4)	35 (38.9)	102 (41.0)	Reference
Yes	202 (59.6)	55 (61.1)	147 (59.0)	1.37 (0.78, 2.43)
Group sex with other men in the past six months				
No	243 (71.5)	59 (65.6)	184 (73.6)	Reference
Yes	97 (28.5)	31 (34.4)	66 (26.4)	2.03 (1.12, 3.68)
Condomless sex with HIV-positive men in the past six months				
No	258 (77.9)	64 (76.2)	194 (78.5)	Reference
Yes	73 (22.1)	20 (23.8)	53 (21.5)	1.56 (0.80, 3.03)
Alcohol use before or during sex with a man in the past six months				
No	143 (41.2)	46 (48.9)	97 (38.3)	Reference
Yes	204 (58.8)	48 (51.1)	156 (61.7)	1.03 (0.58, 1.83)
Recreational drug use before or during sex with a man in the past six months ^b^				
No	207 (59.7)	50 (53.2)	157 (62.1)	Reference
Yes	140 (40.3)	44 (46.8)	96 (37.9)	1.82 (1.05, 3.14)
Current tobacco use in the past 12 months ^c^				
No	89 (25.6)	22 (23.4)	67 (26.5)	Reference
Yes	258 (74.4)	72 (76.6)	186 (73.5)	1.41 (0.76, 2.59)
Alcohol binge in the past 12 months ^d^				
No	100 (28.8)	26 (27.7)	74 (29.2)	Reference
Yes	247 (71.2)	68 (72.3)	179 (70.8)	0.97 (0.55, 1.71)
Recreational drug use in the past 12 months ^b^				
No	66 (19.0)	21 (22.3)	45 (17.8)	Reference
Yes	281 (81.0)	73 (77.7)	208 (82.2)	1.02 (0.53, 1.96)

[^a^] Separate logistic regression models were built to assess correlates of inconsistent follow-up on HIV test results with each behavioral determinant as the primary independent variable. Each model individually adjusted for age, education, annual personal income, and sexual orientation; [^b^] Recreational drug use: self-report intake of rush poppers (alkyl nitrites), crystal meth (methamphetamine), marijuana, hallucinogens (ketamine, LSD, PCP, etc.), cocaine, heroin or other opioids, Magu (a mixture of methamphetamine and caffeine), opium, triazolam tablets (benzodiazepines), or ecstasy (3,4-methylenedioxymethamphetamine, MDMA); [^c^] Tobacco use: intake or smoking (even a puff) the products including regular cigarette, e-cigarette, bidi, cigar, hookah, pipe, dip, chewing tobacco, dissolvable, snuff, or snus; [^d^] Binge drinking: having six or more standard drinks (i.e., 12 ounces (one can) of beer (5% alcohol), 6 ounces (1 glass) of wine (12% alcohol), 1.5 ounces (1 shot) of liquor (40% alcohol)) during a drinking occasion.

**Table 3:** Association of psychosocial factors and inconsistent follow-up on HIV test results among young men who have sex with men in two U.S. cities (N=347).

Characteristics	Total (N = 347) *n* (%)	Not Always (N = 94) *n* (%)	Always (N = 253) *n* (%)	aOR (95% CI) ^a^
Housing stability, median (IQR)	9 (7–10)	8 (5–10)	9 (8–10)	0.87 (0.80, 0.96)
Food insecurity, median (IQR)	0 (0–4)	2 (0–4)	0 (0–3)	1.12 (1.00, 1.26)
Social support, median (IQR)	75 (57–90)	66 (56–76)	76 (58–93)	0.98 (0.97, 0.99)
Resilience, median (IQR)	29 (23–35)	26 (21–31)	30 (24–36)	0.96 (0.94, 0.99)
Internalized homophobia, median (IQR)	5 (4–10)	6 (4–12)	4 (4–9)	1.08 (1.02, 1.15)
Suicidal thoughts/behaviors, median (IQR)	5 (3–8)	4 (3–9)	5 (3–7)	1.02 (0.94, 1.11)
HIV-related stigma, median (IQR)	30 (24–36)	32 (25–36)	30 (24–36)	1.01 (0.98, 1.04)
Loneliness, median (IQR)	19 (16–23)	20 (15–23)	19 (16–23)	1.02 (0.97, 1.08)
Anxiety				
Low risk of anxiety	224 (64.6)	57 (60.6)	167 (66.0)	Reference
High risk of anxiety	123 (35.4)	37 (39.4)	86 (34.0)	1.13 (0.65, 1.94)
Depression				
Low risk of depression	158 (45.5)	40 (42.6)	118 (46.6)	Reference
High risk of depression	189 (54.5)	54 (57.4)	135 (53.4)	1.40 (0.83, 2.37)
Stress				
Low risk of stress	70 (20.2)	6 (6.4)	64 (25.3)	Reference
High risk of stress	277 (79.8)	88 (93.6)	189 (74.7)	4.49 (1.82, 11.12)

[^a^] Separate logistic regression models were built to assess correlates of inconsistent follow-up on HIV test results with each psychosocial determinant as the primary independent variable. Each model individually adjusted for age, education, annual personal income, and sexual orientation.

**Table 4:** Association of inconsistent follow-up on HIV test results and HIV prevention measures among young men who have sex with men in two U.S. cities (N = 347).

HIV Prevention Measures	Total (N = 347) *n* (%)	Not Always (N = 94) *n* (%)	Always (N = 253) *n* (%)	aOR (95% CI) ^a^
PrEP awareness				
No	66 (19.0)	36 (38.3)	30 (11.9)	Reference
Yes	281 (81.0)	58 (61.7)	223 (88.1)	0.37 (0.17, 0.79)
PrEP use (ever)				
No	231 (66.6)	73 (77.7)	158 (62.5)	Reference
Yes	116 (33.4)	21 (22.3)	95 (37.5)	0.82 (0.44, 1.55)
Home testing (ever)				
No	247 (71.2)	63 (67.0)	184 (72.7)	Reference
Yes	100 (28.8)	31 (33.0)	69 (27.3)	1.52 (0.85, 2.74)
HIV testing in the past 12 months				
No	66 (19.0)	38 (40.4)	28 (11.1)	Reference
Yes	281 (81.0)	56 (59.6)	225 (88.9)	0.14 (0.07, 0.27)
HIV testing in the past 6 months				
No	92 (26.5)	41 (43.6)	51 (20.2)	Reference
Yes	255 (73.5)	53 (56.4)	202 (79.8)	0.26 (0.14, 0.45)
HIV testing in the past 3 months				
No	128 (36.9)	48 (51.1)	80 (31.6)	Reference
Yes	219 (63.1)	46 (48.9)	173 (68.4)	0.43 (0.25, 0.73)
HIV testing confidence				
No	197 (56.8)	73 (77.7)	124 (49.0)	Reference
Yes	150 (43.2)	21 (22.3)	129 (51.0)	0.30 (0.16, 0.53)
Condom use self-efficacy				
No	187 (53.9)	60 (63.8)	127 (50.2)	Reference
Yes	160 (46.1)	34 (36.2)	126 (49.8)	0.54 (0.32, 0.92)

[^a^] Separate logistic regression models were built to assess the effects of discontinuity in knowledge of HIV testing status on HIV prevention outcomes with inconsistent HIV test result follow-up as the primary independent variable. Each model individually adjusted for age, education, annual personal income, and sexual orientation.

## Data Availability

Limited de-identified raw data available from the corresponding author upon reasonable request.
